# Match between soaring modes of black kites and the fine-scale distribution of updrafts

**DOI:** 10.1038/s41598-017-05319-8

**Published:** 2017-07-25

**Authors:** Carlos D. Santos, Frank Hanssen, Antonio-Román Muñoz, Alejandro Onrubia, Martin Wikelski, Roel May, João P. Silva

**Affiliations:** 1 Department of Migration and Immuno-ecology, Max Planck Institute for Ornithology, Am Obstberg 1, 78315 Radolfzell, Germany; 2 Núcleo de Teoria e Pesquisa do Comportamento, Universidade Federal do Pará, Rua Augusto Correa 01, Guamá, 66075-110 Belém Brazil; 3 Norwegian Institute for Nature Research, Environmental Data Section, Box 5685 Sluppen, N-7485 Trondheim, Norway; 4 Biogeography, Diversity and Conservation Research Team, Departamento de Biología Animal, Facultad de Ciencias, Universidad de Málaga Spain; 5 Fundación Migres, Ctra. N-340, Km.85, Tarifa, 11380 Cádiz Spain; 6 Department of Biology, University of Konstanz, Universitätsstr. 10, 78464 Konstanz, Germany; 7 REN Biodiversity Chair, CIBIO/InBIO Associate Laboratory, Universidade do Porto, Campus Agrário de Vairão, 4485-661 Vairão, Portugal; 8 CEABN/InBIO – Centro de Ecologia Aplicada “Professor Baeta Neves”, Instituto Superior de Agronomia, Universidade de Lisboa, Tapada da Ajuda, 1349-017 Lisboa Portugal; 9 cE3c – Centro de Ecologia, Evolução e Alterações Ambientais, Faculdade de Ciências da Universidade de Lisboa, Edifício C2, Campo Grande, 1749-016 Lisboa Portugal

## Abstract

Understanding how soaring birds use updrafts at small spatial scales is important to identify ecological constraints of movement, and may help to prevent conflicts between wind-energy development and the conservation of wildlife. We combined high-frequency GPS animal tracking and fine-spatial-scale uplift modelling to establish a link between flight behaviour of soaring birds and the distribution of updrafts. We caught 21 black kites (*Milvus migrans*) and GPS-tracked them while flying over the Tarifa region, on the Spanish side of the Strait of Gibraltar. This region has a diverse topography and land cover, favouring a heterogeneous updraft spatial distribution. Bird tracks were segmented and classified into flight modes from motion parameters. Thermal and orographic uplift velocities were modelled from publically available remote-sensing and meteorological data. We found that birds perform circular soaring in areas of higher predicted thermal uplift and linear soaring in areas of higher predicted orographic uplift velocity. We show that updraft maps produced from publically available data can be used to predict where soaring birds will concentrate their flight paths and how they will behave in flight. We recommend the use of this methodological approach to improve environmental impact assessments of new wind-energy installations.

## Introduction

Miniaturized bio-logging technology suitable to track individual animals in the wild has revolutionized animal ecology as a science, paving the way for “movement ecology” as a new field of research^1^. Since the development of satellite transmitters in the 1980s, a number of technical innovations have made tracking devices smaller, highly precise, longer lasting, and capable of collecting types of information beyond geographical position, such as body motion or heart rate^2, 3^. Similarly, the study of animal movement has evolved from the simple description of trajectories to highly sophisticated inference on behaviour and ecophysiology^2, 4^. Recording Global Positioning System (GPS) data in very high-frequency (>0.1 Hz) is a relatively new capability of tracking devices that has recently boosted the study of avian flight, particularly soaring flight (e.g. refs 5–7).

In general, overland soaring birds show stereotyped flight behaviour responses to uplift variation^8–10^. A critical energetic balance determines the use of soaring or flapping flight. Flapping is energetically costly, and birds use it more often when uplift conditions are not adequate^11–13^. Yet, flapping allows flying in a straight course towards the target, thus promoting faster progression than soaring^14^. Within inland soaring flight, two behavioural modes are commonly observed, slope soaring and thermal soaring^15^. Slope soaring is a response to orographic uplift that forms when horizontal winds are deflected upwards by physical barriers, such as ridges or hills^9, 16^. Birds are able to gain altitude from the windward side of slopes but are also able to soar along ridges disposed linearly, such as mountain ranges^17, 18^. In comparison, thermal soaring is more commonly used in flat areas^9, 17^, but also occurs in steeper terrain^19^. Thermals are formed when low masses of air get in contact with solar exposed terrain, warm-up and rise to several hundreds of meters^16^. Thermals are normally scattered across the landscape, but they may align densely in thermal streets^20^. Thermal soaring is typically divided in two phases, the circling phase where birds climb thermals in a circular ascending trajectory, and the gliding phase, where they achieve horizontal progression by descending in a linear trajectory^20, 21^. While this variety of flight behaviours have been described for some decades from direct observations and radar tracking (e.g. refs 21 and 22), high-frequency tracking has recently helped to better understand the exact manoeuvring during soaring flight^7, 9, 11, 17, 23^. In parallel, much progress has been made in modelling updrafts^12, 16, 19, 24, 25^. In 2012, Bohrer *et al*.^16^ presented an integrative method to produce estimates of orographic and thermal uplift from publically available meteorological and topographical data. This method was later integrated with the track annotation tool of the online database MoveBank (http://www.movebank.org, see ref. 26), which expanded its use in soaring flight studies (e.g. refs 27–30). Although this framework was a remarkable advance for soaring flight research, the low spatial resolution of uplift estimates (0.3° in Bohrer *et al*.^16^ and 0.7° in MoveBank) does not allow us to explore the full potential of high-frequency tracking data. However, understanding how soaring birds use fine-scale updraft distribution is of major relevance to identify ecological constraints of movement^2^, and may help to prevent conflicts between fast-growing wind farming and the conservation of wildlife^31, 32^.

Here we present a spatially explicit approach to identify fine-scale relationships between flight behaviour of soaring birds and updraft spatial distribution. This approach compares soaring modes of birds produced from high-frequency tracking data and fine-scale updraft maps modelled from publically available remote-sensing and meteorological data. Few other soaring bird studies have modelled uplift at a comparable spatial scale^12, 25, 31, 33–36^, and to our knowledge, only three studies combined high-resolution uplift mapping and high-frequency tracking data^25, 35, 36^. Our model species, the black kite (*Milvus migrans*), is an obligate soaring migrant and is among the most abundant soaring birds in the Western European - West African Flyway^37^. We caught and GPS-tracked 21 birds during post-breeding migration in Tarifa (on the Spanish side of the Strait of Gibraltar) while adverse weather conditions at the Strait of Gibraltar prevented them from performing the sea crossing. These circumstances forced the birds to fly around Tarifa for several days. This region has a diverse topography and land cover, favouring a heterogeneous updraft spatial distribution, and thus providing an ideal scenario for the purposes of our study. Bird tracks were segmented and classified into flight modes from motion parameters. Thermal and orographic uplift were mapped using a modified version of the method described by Bohrer *et al*.^16^, that produces higher spatial resolution results. In the case of the orographic uplift, we increased spatial detail by using a 30 m resolution Digital Elevation Model (DEM). For the estimation of thermal uplift, we derived the land surface temperature from a LANDSAT image thermal band following Walawender *et al*.^38^, which produced thermal uplift estimates with a resolution of 100 m. We validated our models by testing the following predictions: (1) birds will adjust their flight behaviour to the updraft spatial distribution, concentrating soaring flight in the areas with higher uplift velocity; and (2) birds will use different soaring modes in areas of thermal or orographic uplift.

## Results

**Figure 1:**
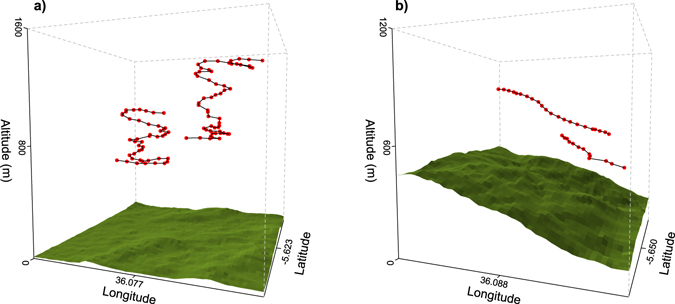
GPS track sections of our dataset illustrating two soaring modes of black kites, (**a**) circling on a flat area (likely thermal soaring), and (**b**) slope soaring. In all tracks birds are moving upwards. GPS fixes (red points) were acquired every 10 seconds. Topography was obtained from a DEM (30 m spatial resolution, released by NASA and the USGS at http://gdex.cr.usgs.gov/gdex/)^60^.

Our tracking dataset included ca. 7580 GPS fixes, after filtering out all non-flight sections. The resolution of the GPS data allowed for a clear identification of the soaring modes of birds (Fig. 1). Birds took anywhere from tens of seconds to minutes to complete ascending circles during circular soaring, with each circle being clearly discriminated in the resolution of our dataset (Fig. 1a). Also flight sections where birds exhibited slope soaring were documented by tens of fixes, reflecting an overall linear trajectory (Fig. 1b).

**Figure 2:**
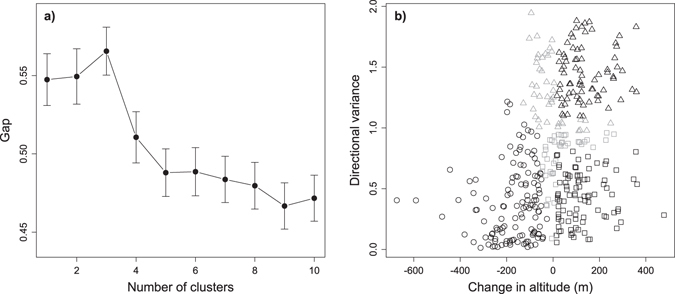
(**a**) Gap statistic as a function of the number of clusters. Clusters were built from the variables elevation change and directional variance with the K-means algorithm. Error bars represent standard error. The optimal number of clusters for this dataset stands on three clusters, i.e., the first maximum that does not overlap with the standard error of earlier observations (see Maechler *et al*.^58^). (**b**) Distribution of track segments according their elevation change and directional variance values, and classified in three clusters (different symbols) as defined in (**a**). Circles, triangles and squares represent, gliding, circular soaring and linear soaring track segments, respectively. Grey observations, placed at the boundaries of clusters, were excluded from further analysis in order to define flight modes more distinctively. These were gliding segments with elevation change above −15 m, circular and linear soaring segments with elevation change below 15 m (these representing 15% of all track segments), and 15% of the remaining segments that stand at the boundary between circular and linear soaring regarding directional variance.

Gap analysis showed that our track segments were optimally divided into three clusters, when characterized from their elevation change and directional variance (Fig. 2a). A first cluster was characterized by negative elevation change and low directional variance (circles in Fig. 2b), as expected in gliding behaviour. Both the other two clusters showed positive elevation change and either high directional variance (triangles in Fig. 2b) or low directional variance (squares in Fig. 2b), reflecting circular and linear ascending flight behaviours respectively. Observations placed at the boundaries of clusters (grey points in Fig. 2b) were excluded from further analysis in order to define the flight behaviours more distinctively.

**Figure 3:**
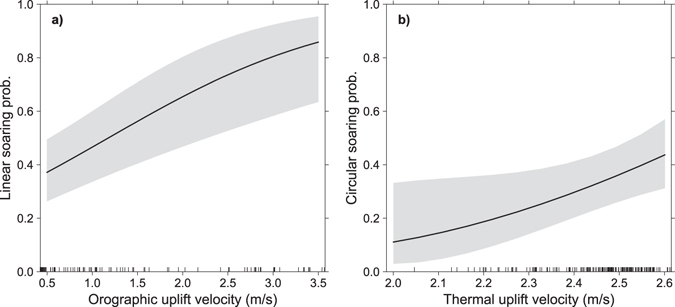
Model effects of orographic and thermal uplift on soaring modes of black kites. (**a**) Partial effect of orographic uplift velocity on linear soaring, and (**b**) partial effect of thermal uplift velocity on circular soaring. (**a**) and (**b**) resulted from two binomial GLMM models. In (**a**) the response variable was 1 if the track segment was classified as linear soaring and 0 if classified as gliding. Model fixed factors were orographic and thermal uplift velocities, and random factors were bird identifier and the day of data collection. In (**b**) the model was similar to (**a**) but dependent variable was 1 for circular soaring and 0 for gliding. Only factors with significant effect in the models are shown. Shading represents 95% confidence intervals.

**Figure 4:**
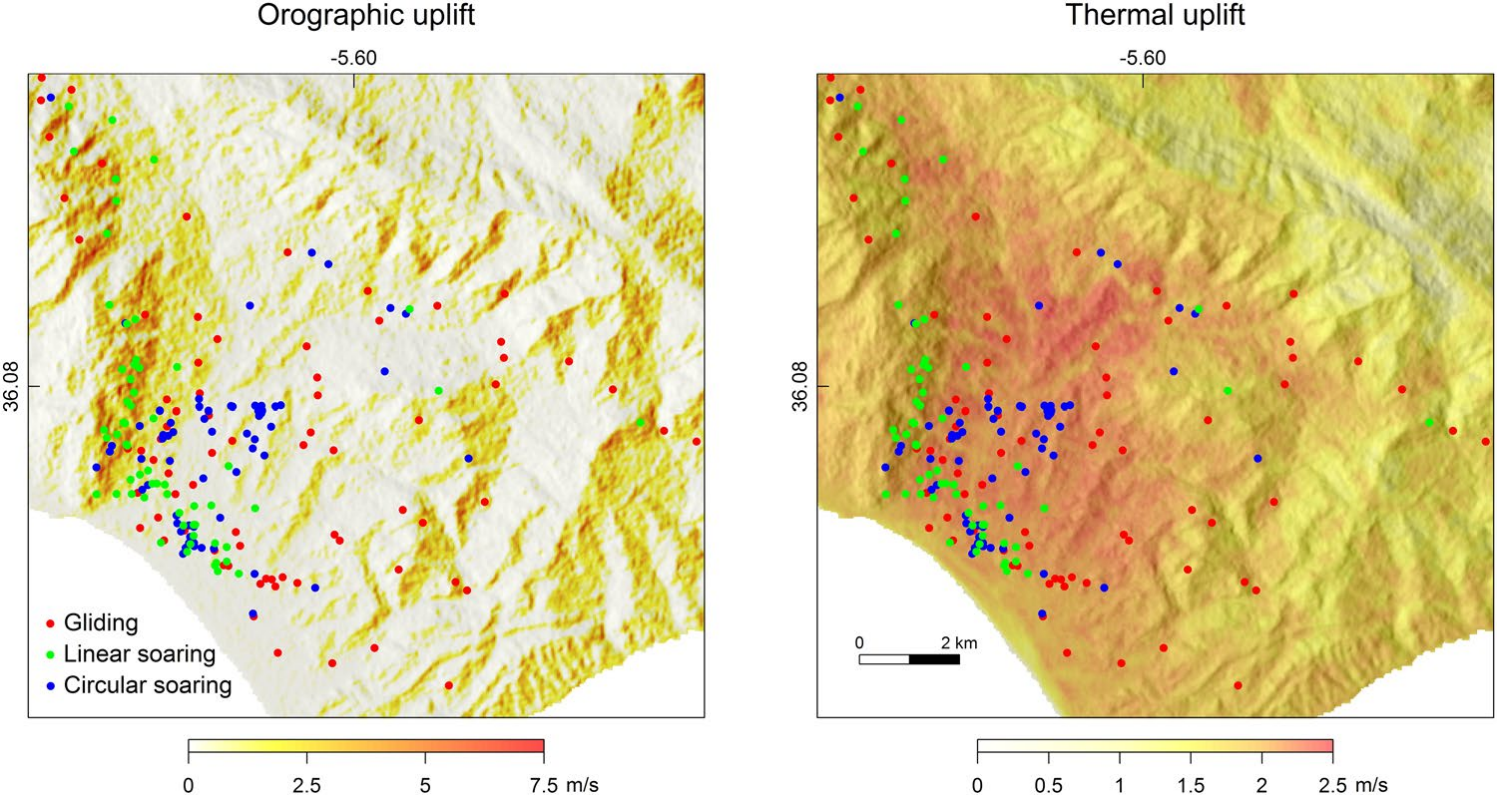
Correspondence between the uplift distribution and black kites flight modes (coloured dots). Only the part of the study area with higher bird use is shown. Orographic lift reflects levanter wind (direction: mean = 115.7°, SD = 0.02, range = 112–119; speed: mean = 10.4 m s-1, SD = 1.49, range = 3.7–12.7), when most of our tracking data was collected. Dots are positioned at the centroid of the respective track segment. Uplift maps are coloured from white to orange as higher uplift velocities were estimated (see methods). Light hill shading was added to illustrate interaction between topography and uplift. Uplift models used a LANDSAT image available at the USGS archive (http://earthexplorer.usgs.gov/)^64^, a DEM released by NASA and the USGS (at http://gdex.cr.usgs.gov/gdex/)^60^ and were developed with the software ArcGIS^61^ (see methods section for further details).

Orographic uplift velocity had a significant positive effect on the probability of birds to display linear soaring, and thermal uplift had a similar effect on circular soaring (Table 1 and Fig. 3). Linear soaring occurred more often on the windward side of mountain slopes, where orographic uplift was estimated higher, while circular soaring was more frequent on valleys and visually matched with areas of higher estimated thermal uplift (Fig. 4). It should be noted that the contribution of thermal uplift for linear soaring probability was near statistical significance (Table 1).

**Table 1:** Summary of binomial GLMMs testing the effect of orographic and the thermal uplift velocities (m/s) on soaring modes.

Parameter	Estimate	SE	*Z*	P-value	R^2^ cond./marg.
Model 1: Circular soaring					0.14/0.04
Intercept	−8.16	3.42	−2.38	0.017	
Thermal uplift velocity	3.04	1.36	2.23	0.026	
Orographic uplift velocity	0.03	0.25	0.10	0.918	
Model 2: Linear soaring					0.12/0.12
Intercept	−6.64	3.17	−2.10	0.036	
Thermal uplift velocity	2.33	1.23	1.90	0.058	
Orographic uplift velocity	0.78	0.19	4.04	<0.001	

The response variable was 1 if the track segment was classified as linear soaring or circular soaring (depending on the model) and 0 if classified as gliding. Model fixed factors were orographic and thermal uplift velocities, and random factors were bird identifier and the day of data collection. Conditional and marginal R^2^ were calculated following Nakagawa and Schielzeth^66^.

Climbing speed was not statistically different between soaring modes (Table 2). Ground speed was significantly higher in linear soaring, and birds flew at higher altitudes above the ground level when performing circular soaring (Table 2). The tailwind component (velocity vector component in the direction of the wind) was high during linear soaring indicating that the birds moved, at least partially, in the direction of the wind (Table 2). During glides, the low tailwind component and high horizontal displacement showed that birds moved in straight trajectories regardless of the wind direction (Table 2). No differences were found between soaring modes on the dorso-ventral acceleration amplitude (proxy of flapping, Table 2), with average values comparable to that of glides and about a half of that expected for flapping flights (see methods).

**Table 2:** Summary of parameters of the different flight modes.

Flight parameter	Gliding	Circular soaring	Linear soaring	P-value	R^2^ cond./marg.
Vertical speed (m s^−1^)	−0.83 ± 0.049 (129)	0.66 ± 0.054 (75)	0.61 ± 0.053 (90)	0.45	—
Ground speed (m s^−1^)	9.7 ± 0.30 (129)	5.4 ± 0.14 (75)	7.3 ± 0.25 (90)	<0.001	0.38/0.11
Horiz. displacement (m)	1685 ± 65.4 (129)	363 ± 23.3 (75)	1209 ± 52.7 (90)	—	—
AGL (m)	428 ± 21.8 (129)	463 ± 32.5 (75)	342 ± 23.3 (90)	0.012	0.15/0.04
Tailwind component	0.04 ± 0.057 (129)	−0.01 ± 0.027 (75)	0.42 ± 0.056 (90)	—	—
D-v accel. amplitude (g)	0.15 ± 0.007 (90)	0.16 ± 0.009 (56)	0.15 ± 0.008 (68)	0.74	—

Parameters are summarized as mean ± standard error (sample size). P-values compare only the two soaring modes, and result from GLMM models where the flight parameter is the response variable, the soaring model is the fixed factor and bird identity and the day of data collection are the random factors. Conditional and marginal R^2^ were calculated following Nakagawa and Schielzeth^66^.

## Discussion

We showed that soaring modes of black kites are well explained by the fine-scale updraft distribution. Birds showed higher probability of performing circular climbing in areas of higher predicted thermal uplift, while linear climbing was more likely to happen in areas of higher predicted orographic uplift velocity. These results confirm our predictions and are in accordance with the literature on this topic (e.g. refs 9, 10 and 17). Importantly, our models show that fine-resolution uplift maps produced from publically available remote-sensing and meteorological data can be used to map the flight behaviour of soaring birds. Similar approaches were used in earlier studies to infer flight behaviour^12, 25, 34^ and space use^31, 33^ of different soaring bird species. It should be noted that our models include a large amount of unexplained variance, although this is also reflected in previous studies linking soaring bird flight behaviour to uplift proxies^12, 16, 27, 29^. Part of this variability should be natural, but we must emphasize that all uplift estimators used in the literature are reasonably imprecise^16^. However, this does not decrease the value of our models to predict general space use and flight behaviour of soaring birds, particularly for wildlife management purposes. In our study, uplift maps were temporally generic, which could explain some of the model variance. For orographic uplift we modelled two generic wind conditions occurring during the tracking data collection (strong levanter and light western breeze), but did not account for small temporal scale variations within those conditions. In the thermal uplift modelling, we derived land surface temperature from a LANDSAT image acquired approximately one year before the tracking data collection began, for the reasons explained in the methods. Although we showed a high stability of between-year thermal uplift (Supplementary Table S1), some variation in thermal intensity is expected between days, due to weather variability, and within a day, due to solar radiation variability^39^. Altogether, these aspects may have promoted some degree of mismatch between the uplift models and the bird behaviour.

One unexpected result was the use of linear soaring in areas close to the seashore, far from areas with orographic uplift (see Fig. 4). We further investigated the characteristics of these flights and found that they only differ from inland linear soaring flights in dorso-ventral acceleration amplitude and ground speed (Supplementary Table S2). Dorso-ventral acceleration amplitude was higher in the coastal linear soaring flights, indicating higher probability of flapping. However, as for the remaining flights, the magnitude of this parameter was still far from that expected for flapping flights (see methods). Therefore, flapping did not explain the linearity of these coastal flights. Regarding ground speed, coastal linear soaring flights were slower than the remaining linear soaring flights, with speeds comparable to circular soaring (Table 2, Supplementary Table S2). We should also note that thermal uplift had a marginally significant effect on linear soaring probability in our models (Table 1), which was most likely caused by these coastal linear flights. These facts seem to indicate that birds flying linearly near the coastline were using a thermal street. Such behaviour has been described before in other soaring birds^21, 40^.

When comparing parameters of the two flight modes, we found that circular and linear soaring had similar climbing speeds despite the fundamental differences in other parameters (Table 2). This illustrates well the high behavioural flexibility of this species in efficiently exploring a diversity of uplift conditions. A notable aspect of linear soaring was its high tailwind component, showing that flight direction was at least partly directed towards slopes with uplift (Table 2). This behaviour may help birds to gain altitude in the small spatial scale. However, it does not prevent birds to move along slopes, as it has been reported in earlier studies^17, 18^. In contrast, gliding had an overall low tailwind component despite of their linear path (Table 2). This is in agreement with earlier knowledge indicating that gliding directions are chosen based on a combination of destination goals and prevailing winds^41, 42^. We should also emphasize that flapping was very improbable in our flights given the low values of dorso-ventral acceleration amplitude recorded. This is in accordance with other studies showing that large and medium sized soaring raptors rarely use flapping flight^11, 43–46^.

In comparison with earlier uplift mapping methods, ours shares with that of Bohrer *et al*.^16^ the advantage of using publically available data, thereby facilitating use in science and wildlife management. Our thermal uplift mapping method improves that of Bohrer *et al*.^16^ by producing fine-scale estimates of uplift. This becomes possible when estimating land surface temperature from LANDSAT imagery. The full LANDSAT series is publically available at http://earthexplorer.usgs.gov/, containing images since 1972 all around the globe. However, scenes are only acquired every 16 days and at the same time of the day, which might be limiting for some applications. Similarly, our orographic uplift mapping method produces higher resolution maps of uplift than that of Bohrer *et al*.^16^ through the use of a higher resolution DEM. This DEM is available for the whole world at http://gdex.cr.usgs.gov/gdex/. However, the overall accuracy of this uplift model also depends on how detailed the wind data is. For large areas it is convenient that data from different weather stations is used, because wind patterns may change geographically. In addition, it is important to consider the temporal resolution of wind data, as wind conditions may change significantly within short timeframes thus affecting the orographic uplift estimates. We should also note that many regions in the world are not monitored by weather stations. For those regions we recommend the use wind data from global atmospheric models, such as the ECMWF available in MoveBank (http://www.movebank.org).

We have shown that flight behaviour of soaring birds can be inferred from fine-scale uplift maps, and this may have relevant applicability to prevent collisions between soaring birds and vertical anthropogenic structures. In particular, wind-energy installations can have major impacts on soaring bird populations^47, 48^, with collision rates surpassing one bird per turbine per year in some areas^49^. However, even among closely located wind farms collision rates may be dramatically different^49, 50^. Fine-scale updraft distribution may be a key factor determining these differences. As we have shown here, areas with higher uplift potential concentrate the bird’s flight activity, and the type of uplift will determine soaring behaviours. Additionally, certain flight behaviours are more likely to cause collisions with wind turbines than others^32, 50, 51^. Unfortunately, this evidence is rarely considered in environmental impact assessments (EIA) of new wind-energy installations^49^, which is likely due to a lack of pragmatic methods to predict soaring bird’s landscape use. We hope that our updraft mapping method contributes to fill this gap and ultimately help in the conservation of soaring birds.

## Methods

### Ethical note

The experimental procedures of this study, including bird trapping and the GPS tagging, were conducted according to Spanish regulations on animal welfare and experimentation, and were approved by the Consejería de Medio Ambiente of the Junta de Andalucía through the license to Alejandro Onrubia.

### Tracking data collection

This study was conducted in Tarifa (36.0132°N, 5.6027°W), on the Spanish side of the Strait of Gibraltar. The Strait of Gibraltar is a well-known migratory bottleneck for soaring birds, which are unable to cross wide bodies of water and gather in large numbers at narrow sea passages^37^. During the summer months, when the post-breeding migration takes place, high-speed levanter winds (10–20 m/s from the east) are very common in this area^52^. These winds can persist for periods up to a week^52^, blocking the passage of soaring birds to Africa and causing large accumulations around Tarifa^53^. The black kite is the most common soaring bird during the post-breeding migration and was shown to be particularly affected by levanters at the strait^53^. Congregations of several thousands were observed around Tarifa during levanters, while this study was conducted. We captured and GPS tracked 21 black kites during a levanter period, aiming to describe their flight behaviour while using the region around Tarifa. This area has a complex topography, with high mountain ranges (up to 700 m) and dry stony valleys, creating a heterogeneous thermal and orographic updraft spatial distribution (see Fig. 4). Birds were captured on August 20th 2012, using a walk-in trap (7 × 7 × 3.5 m) baited with carrion, located 3.5 km North of Tarifa. GPS-GSM data loggers (42 g, TM-202/R9C5 module, Movetech, UK, https://www.uea.ac.uk/movetech) were attached to the birds as backpacks using Teflon harnesses. A stretched loop of rubber band (less than 5 mm long) was placed between the data logger and each of the four harness endings to serve as a weak-link. Previous tests of this method showed that the rubber band breaks within two to four weeks when exposed solar radiation causing drop-off. Birds were released a few hours after capture, immediately after tagging, and were tracked for the next three days. Data loggers obtained GPS positions (with horizontal and vertical mean error of 1.5 m) every 10 seconds and 20-second bursts of 1 Hz acceleration every 3 minutes. This information was sent remotely, through the GSM network, to an internet server. Wind parameters were measured every 10 minutes from a weather station located in Tarifa (36.0138°N, 5.5988°W).

### Estimation of flight parameters and definition of flight modes

Sections of continuous flight during daylight hours (8 am to 7:30 pm, local time) were selected from the original tracking data. These sections were further divided into segments of 200 seconds for which we estimated the following flight parameters:

1. AGL (m) – mean altitude above ground level.
2. Horizontal displacement (m) – horizontal distance between the first and last position in the segment.
3. Elevation change (m) – height distance between the first and last position in the segment.
4. Ground speed (m s^−1^) – average of velocity vector scalars calculated for consecutive positions in the track segment.
5. Vertical speed (m s^−1^) – calculated as ground speed but in the vertical axis.
6. Directional variance – angular variance of velocity vector directions in the track segment. Velocity vector directions were calculated for consecutive positions in the track segment.
7. Tailwind component – velocity vector component in the direction of the wind. Velocity vector here is the mean vector of all velocity vectors calculated for consecutive positions in the track segment. Wind direction was sampled from the weather station every 10 minutes. Therefore the time lag between the GPS recording of the track segment and the recording of the wind direction at the weather station was never more than 5 minutes.
8. Dorso-ventral acceleration amplitude (g) – average of acceleration heave amplitudes. Heave amplitudes are the range between each heave value and the average of the heave values in the track segment. This variable was used as a proxy of flapping behaviour^54^. The flapping flight reference value of this variable (mean = 0.30, standard error = 0.015) was obtained from video recordings of four tagged birds as they took-off.

GPS misreadings (repeated or missing coordinates) were deleted from datasets, and track segments with less than ten positions were excluded from analysis (3% of the original dataset). Also, for 25% of our track segments acceleration values were misread or we had less than ten readings. In those cases, dorso-ventral acceleration amplitude was not calculated. All the flight parameter calculations were done with the software R^55^, and the estimation of circular parameters used the package circular^56^.

Elevation change and directional variance were the parameters chosen to classify flight modes. Elevation change has the potential to segregate gliding from soaring, depending if the bird climbed or dropped during the track segment. On the other hand, the directional variance values of climbs showed a bimodal distribution (see Supplementary Fig. S1), suggesting the existence of two soaring modes that differ in track sinuosity. The remaining flight parameters were also examined for their potential to segregate flight modes but they were not utilized. Flight modes were objectively identified from elevation change and directional variance using the gap statistic^57^, implemented in R^55^ by the function clusgap of the package cluster^58^. This technique identifies the optimal number of clusters when applied to multiple clustering outputs of the same dataset. Specifically, the gap value compares the change in within-cluster dispersion with that expected under a reference null distribution (defined via bootstrap) for a given number of clusters^57^. The curve of the gap value as a function of the number of clusters allows selection of the optimal number of clusters (see Fig. 2a). The clusgap function was set with the K-means clustering algorithm, a maximum of ten clusters, and 1000 bootstrap replications. The optimal number of clusters was defined as the first maximum that does not overlap with the standard error of earlier observations (the default method set in Maechler *et al*.^58^).

### Estimation of orographic and thermal uplift

The orographic uplift velocity (*w*_0_), caused by the interaction between horizontal wind at ground level and topography, was estimated as suggested by Bohrer *et al*.^16^ and Brandes and Ombalski^59^ from the following equations:

1 $${w}_{0}=v\ast {C}_{\alpha }$$

2 $${C}_{\alpha }=\,\mathrm{Sin}(\theta )\ast \,\mathrm{Cos}(\alpha -\beta )$$

where *v* is the horizontal wind speed (m s^−1^), ${C}_{\alpha }$ is the updraft coefficient, *α is* the horizontal wind direction at ground level (in degrees, North = 0), *β* is the terrain aspect (in degrees, North = 0) and *θ* is the terrain slope angle (in degrees).

Measurements of *v* and *α* were derived from the weather station data. We modelled the orographic uplift for the two main wind conditions observed during the sampling period: strong levanter (wind direction: mean = 115.7°, SD = 0.02, range = 112–119°; wind speed: mean = 10.4 m/s, SD = 1.49, range = 3.7–12.7 m/s) and light western breeze (wind direction: mean = 284.6°, SD = 0.20, range = 257–295°; wind speed: mean = 3.1 m/s, SD = 0.38, range = 2.5–3.7 m/s). We should emphasise that within each of these two conditions wind parameters were highly stable, therefore the two orographic uplift models represented well what was experienced by the birds. Topography variables, *β* and *θ*, were calculated from a DEM (30 m spatial resolution)^60^, made available by the National Aeronautics and Space Administration (NASA) and the United States Geological Survey (USGS) at http://gdex.cr.usgs.gov/gdex/, using ArcGIS^61^ Slope and Aspect functions of the Spatial Analyst extension. The updraft coefficient (${C}_{\alpha }$) varies between −1 and 1, where negative values represent the leeward side of the terrain and positive values its windward side. Negative ${C}_{\alpha }$ values reflect turbulent eddies and lee waves, and were set to 0 for simplicity. We also did not correct *w*_0_ for terrain channelling and wind sheltering effects.

The thermal uplift velocity (${w}^{\ast }$) is due to the vertical air flux caused by the diurnal solar heating of the continental earth surface. The natural circumstances of this phenomenon include high levels of turbulence that are not considered here. Following Bohrer *et al*.^16^, ${w}^{\ast }$ can be expressed as:

3 $${w}^{\ast }={[\frac{g\ast z\ast H}{\theta }]}^{1/3}$$

where *g* is the gravitational acceleration (9.8 m s^−2^), *z* is the bird’s flight height (set as the average height in meters of all GPS data obtained during active flight), *H* is the surface sensible heat flux (W m^−2^) and *θ* is the potential temperature (K).

The surface sensible heat flux (*H*) is the process where heat energy is transferred from the terrain surface to the atmosphere through conduction and convection, expressed by Hu *et al*.^62^ as follows:

4 $$H=p\ast {c}_{p}\frac{({T}_{s}-{T}_{a})}{{r}_{a}}$$

where *p* is the air density at sea level and 288.15 K temperature (1.225 kg m^−3^), *c*_*p*_ is the isobaric mass heat capacity of sea level dry air at 273.15 K (1.0035 J kg^−1^ K^−1^), *T*_*s*_ is the land surface temperature, *T*_*a*_ is the air temperature 2 m above ground level (288.15 K) and *r*_*a*_ is the aerodynamic resistance for a grassland surface (formulated by Allen *et al*.^63^ as 208 divided by the horizontal wind speed at 2 meters height). The land surface temperature (*T*_*s*_) was calculated from a LANDSAT image thermal band (100 m spatial resolution) in ArcGIS^61^ as described in Walawender *et al*.^38^. Because LANDSAT 7 ETM + images from the summer 2012 (period of tracking data collection) have data gaps due to a scan line corrector failure, we used a LANDSAT 8 OLI/TIRS image acquired in the following summer (17 July 2013, 11:58 am local time)^64^. However, this has no relevant consequences in our results since the thermal uplift estimates obtained from different images of the summers of 2012 and 2013 are highly correlated (see Supplementary Table S1). LANDSAT images were downloaded from the USGS archive (http://earthexplorer.usgs.gov/).

The potential temperature (*θ*) is the temperature of an unsaturated part of dry air if brought adiabatically and reversibly from its initial state to a standard pressure, expressed by Stull^39^ as follows:

5 $$\theta =T{(\frac{{p}_{0}}{p})}^{k}$$

where *T* is the air temperature 2 m above ground level (set to 288.15 K), *p*_0_ is the sea level standard air pressure (1013.25 mbar), *p* is the atmospheric boundary layer air pressure 1 km above sea level (898.7457 mbar) and *k* is the Poisson constant for dry air (0.2854).

### Modelling of soaring modes

The effects of orographic and thermal uplift on soaring modes were modelled with binomial Generalized Linear Mixed Models (GLMM), using the function glmer of the R package lme4^65^. We built separate models for linear soaring and circular soaring. The response variable was assigned as 1 if the track segment was classified as linear soaring or circular soaring (depending on the model), and 0 if classified as gliding. Glides were used as null observations of soaring because they do not depend on uplift (see Fig. 4). The model’s fixed factors were the orographic and the thermal uplift velocities and the random factors were bird identity and the day of data collection. Random factors controlled for the fact that different track segments of the same bird were not independent, and track segments obtained during the same day may also have a degree of dependency. Two extreme observations of orographic uplift (4.6 and 5.1 m/s) that caused convergence problems in the models were removed from the dataset. Flight parameters of the two soaring modes were also compared using GLMM, but fitted for Gaussian distributions with the function lmer of the R package lme4. Different models were adjusted for each of the flight parameters compared. Models were not produced for horizontal displacement and tailwind component because the comparisons of these parameters between flight modes were not deemed biologically relevant. In the models, the response variable was the flight parameter and the fixed factor was the soaring mode (linear soaring or circular soaring). Random factors were bird identity and the day of data collection, as in earlier models. AGL and ground speed were log-transformed to normalize their distributions. Goodness-of-fit was evaluated for all models through marginal R^2^ (variance explained by the fixed effects) and the conditional R^2^ (the variance explained by the fixed and random effects) following Nakagawa and Schielzeth^66^.

## Electronic supplementary material

Supplementary information
