# Supplementary material for: Match between soaring modes of black kites and the fine-scale distribution of updrafts
Source: Sci Rep. 2017 Jul 25;7:6421. doi: 10.1038/s41598-017-05319-8 (PMC5526945; doi:10.1038/s41598-017-05319-8)
Supplement: Supplementary file 1 — Supplementary information [file 41598_2017_5319_MOESM1_ESM.pdf]

Santos, C.D., Hanssen, F., Muñoz, A., Onrubia, A., Wikelski, M., May, R. & Silva, J.P.

Match between soaring modes of black kites and the fine-scale distribution of updrafts

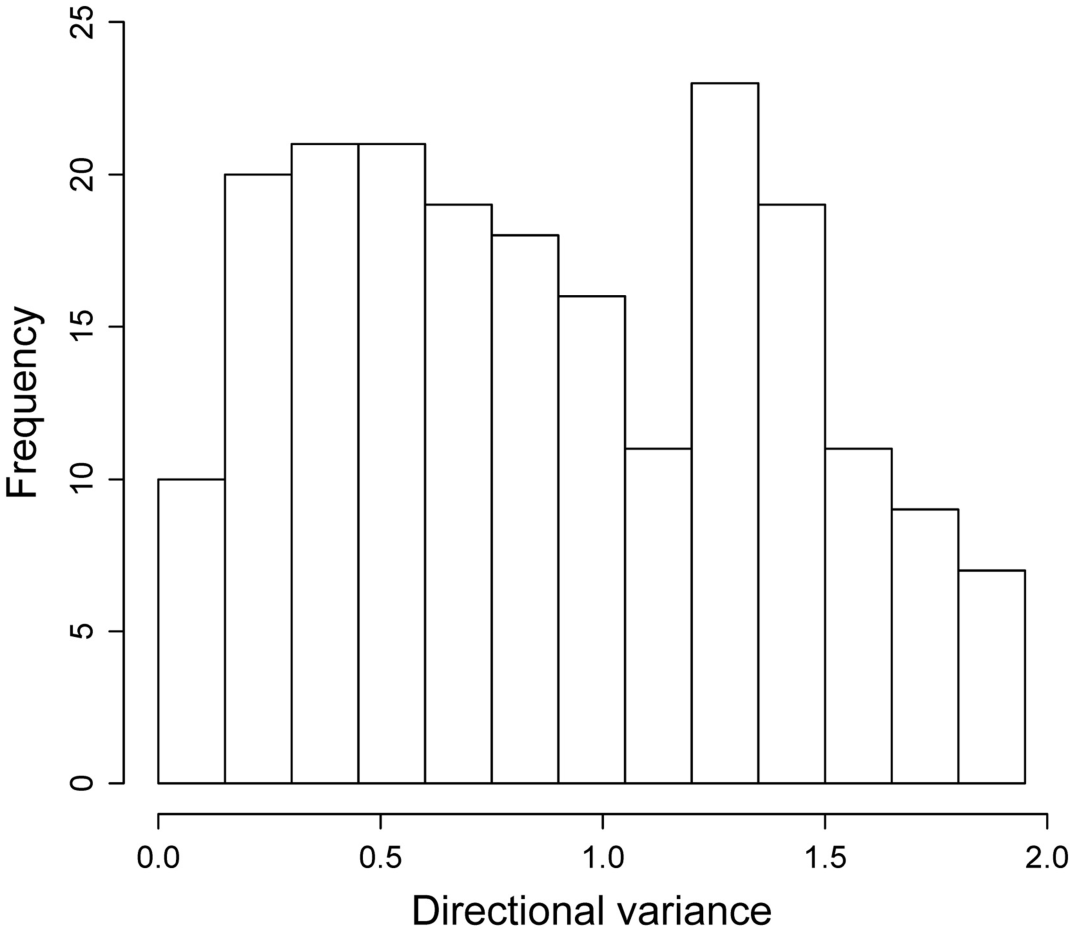

Supplementary Figure S1. Distribution of the track segments with positive elevation change regarding their directional variance.

Supplementary Table S1. Correlation matrix of thermal uplift velocity (m/s) estimates obtained from different LANDSAT images[1-5]. All LANDSAT images were acquired in the summer. Acquisition dates are shown in the dd/mm/yyyy format.

	06/07/2012	15/09/2012	08/07/2013	10/09/2013	17/07/2013
06/07/2012	1	0.88	0.85	0.86	0.77
15/09/2012	0.88	1	0.89	0.92	0.92
08/07/2013	0.85	0.89	1	0.90	0.94
10/09/2013	0.86	0.92	0.90	1	0.94
17/07/2013	0.77	0.92	0.94	0.94	1

Supplementary Table S2. Summary of parameters of linear soaring near the sea coast and farther inland. Flights considered near the sea coast were those positioned within 2.5 km from the sea edge, and at least 1 km far from mountains or hills that could provide orographic uplift. Parameters are summarized as mean ± standard error (sample size). P-values result from GLMM models with the flight parameter as the response variable, the linear soaring category as fixed factor and bird identity and the day of data collection as random factors. AGL, elevation change, horizontal displacement, ground speed and tailwind component were log-transformed to normalize their distributions. Conditional and marginal R^2 were calculated following Nakagawa and Schielzeth[6].

Flight parameter	Coastal linear soaring	Inland linear soaring	P-value	R^2 cond./marg.
AGL (m)	349 ± 52.5 (21)	340 ± 26.0 (69)	0.20	-
Directional variance	0.48 ± 0.035 (21)	0.41 ± 0.025 (69)	0.33	-
Horiz. displacement (m)	1005 ± 92.2 (21)	1271 ± 61.2 (69)	0.17	-
Ground speed (m s^{-1})	6.1 ± 0.47 (21)	7.7 ± 0.29 (69)	0.033	0.24/0.06
Elevation change (m)	102 ± 22.3 (21)	130 ± 11.2 (69)	0.22	-
Vertical speed (m s^{-1})	0.52 ± 0.114 (21)	0.63 ± 0.060 (69)	0.39	-
Tailwind component	0.10 ± 0.142 (21)	0.52 ± 0.055 (69)	0.90	-
D-v accel. amplitude (g)	0.19 ± 0.022 (16)	0.14 ± 0.008 (52)	0.018	0.11/0.07

References

1. NASA Landsat Program. *Landsat OLI/TIRS scene LC82010352013198LGN00, L1T, USGS, Sioux Falls, 17/07/2013* (2015).
2. NASA Landsat Program. *Landsat ETM+ scene LE72010352012188ASN00, L1T SLC-Off, USGS, Sioux Falls, 06/07/2012* (2014).
3. NASA Landsat Program. *Landsat ETM+ scene LE72020352012259EDC00, L1T SLC-Off, USGS, Sioux Falls, 15/09/2012* (2014).
4. NASA Landsat Program. *Landsat OLI/TIRS scene LC82020352013189LGN00, L1T, USGS, Sioux Falls, 08/07/2013* (2015).
5. NASA Landsat Program. *Landsat OLI/TIRS scene LC82020352013253LGN00, L1T, USGS, Sioux Falls, 10/09/2013* (2015).
6. Nakagawa, S. & Schielzeth, H. A general and simple method for obtaining R^2 from generalized linear mixed-effects models. *Methods Ecol. Evol.* **4** (2013).
